# Suicide prevention training for pharmacy students in Ireland: A pre-post survey evaluation

**DOI:** 10.1016/j.rcsop.2026.100839

**Published:** 2026-09-01

**Authors:** Michelle O'Driscoll, Kerrie Gallagher, Maria Donovan, Laura Sahm, Aoife Fleming, Clíodhna O'Brien, Ailish O'Neill, Eve Griffin

**Affiliations:** a National Suicide Research Foundation, Cork, Ireland; b School of Pharmacy, University College Cork, Cork, Ireland; c Health Service Executive, National Office for Suicide Prevention, Dublin, Ireland; d School of Public Health, University College Cork, Cork, Ireland

**Keywords:** Uicide prevention, Competency, Pharmacy education, Curriculum

## Abstract

**Background:**

Pharmacists regularly encounter individuals experiencing suicidal distress. However, suicide prevention education is not routinely offered to pharmacy students. This study evaluated (i) the impact of a suicide prevention workshop on students' attitudes and perceived competence in suicide prevention and; (ii) the feasibility of embedding such training into pharmacy curricula.

**Methods:**

A pre-post survey was conducted with students enrolled in the final year of a Pharmacy programme in Ireland. A three-hour suicide prevention workshop was delivered as part of their Clinical Pharmacy module. Workshop content included suicide epidemiology, risk and protective factors, communication and safety planning, and student personal wellbeing. Quantitative outcomes measured included changes in perceptions of suicide prevention importance and competence and workshop acceptability, feasibility, and appropriateness.

**Results:**

Of the 53 students who attended the workshop, 26 students completed the evaluation. Students' perceived suicide prevention importance scores increased post-workshop (mean difference = 1.82, *p* = 0.004). Perceived competence scores also increased (mean difference = 15.65, *p* < 0.001). Post-workshop perceptions were positive, with mean scores of 28.81/30 for acceptability, 13.56/15 for feasibility, and 21.78/25 for appropriateness. Students supported continued inclusion of the content within the programme, with most indicating a preference to receive such training earlier.

**Conclusions:**

This study demonstrates that suicide prevention education improves pharmacy students' perceived readiness to respond to individuals at risk of suicide. The workshop was successfully embedded within the pharmacy curriculum, and viewed positively by students, who strongly supported continued curricular inclusion. Suicide prevention education represents an important consideration for current and future pharmacy training.

## Highlights

• Pharmacy students' suicide-prevention skills improved after a three-hour workshop.
• The suicide prevention workshop was rated as being highly feasible, acceptable, and appropriate.
• Students supported the embedding of suicide prevention training into the pharmacy curriculum.

## 1 Introduction

Suicide is an international public health concern, accounting for more than 700,000 deaths annually.^1^ Beyond mortality, suicide and self-harm place substantial emotional, social and economic burdens on individuals, families, communities and healthcare systems.^2^, ^3^ As many individuals who die by suicide have contact with healthcare systems in the months leading up to their death,^4^, ^5^ healthcare professionals (HCPs) are recognised as having an important role in early identification, compassionate engagement, and signposting to the appropriate supports.

Pharmacists are among the most accessible HCPs, engaging in frequent contact with the public across primary, secondary and tertiary care settings.^6^ Community pharmacists may encounter individuals experiencing physical or mental health difficulties, substance misuse, chronic pain, or social inequalities, all of which can be associated with an elevated suicide risk.7., 8, 9 Pharmacists also regularly interact with patients prescribed psychotropic medications, individuals presenting following self-harm, and/or family members seeking advice and support.^6^ As highly trusted professionals,^10^ pharmacists are uniquely positioned to adopt a gatekeeper role in suicide prevention, contributing to the early identification of risk, engaging individuals in supportive conversations, and facilitating timely referral to appropriate services.^11^ However, previous studies suggest that both pharmacy students and practising professionals report low confidence and limited preparedness in supporting those at risk of suicide, despite recognising suicide prevention as an important aspect of care provision.^8^, ^12^

There is increasing international recognition that suicide prevention education should be embedded across health and social care curricula to better prepare future professionals for patient-facing practice.^13^, ^14^, ^15^ International evidence indicates that suicide prevention training can improve learners' attitudes, self-efficacy, and willingness to engage with individuals experiencing suicidal distress.^13^ Within pharmacy, studies from several countries have explored the impact of suicide prevention education for pharmacy students.^16^, ^17^, ^18^, ^19^ For example, in the United Kingdom an interprofessional suicide awareness training for mental health nursing and pharmacy students improved self-reported preparedness for suicide prevention in both groups, although mental health nursing students reported higher preparedness overall.^18^ Similarly, studies exploring the impact of training on practicing pharmacists reflect growing recognition of their potential role in suicide prevention practice.^17^, ^20^, ^21^ Overall, findings suggested that such training has the potential to improve pharmacists attitudes, and perceived readiness to respond to those at risk.^16^, ^17^, ^21^ However, much of this work has focused solely on learner outcomes, with less known about how students perceive such training in relation to its acceptability, feasibility, and appropriateness. These implementation outcomes are important as educational interventions that demonstrate positive effects may nonetheless be difficult to sustain or scale if they are not perceived as relevant or suitable within a curriculum.

Furthermore, to our knowledge, there is currently no published evaluation of structured suicide prevention education for pharmacy students in Ireland. Given the important, accessible, and trusted role of pharmacists within Irish healthcare, and the increasing emphasis on mental health and suicide prevention policy nationally,22., 23. evidence is needed regarding the integrating of such training within Irish pharmacy education.

### 1.1 Study aim

The aim of this study was to determine:

(i) the impact of an embedded three-hour workshop on pharmacy students' suicide prevention attitudes and competence and;
(ii) Students' perspectives on the workshop's acceptability, feasibility, and appropriateness.

## 2 Methods

### 2.1 Study design

This study employed a pre-post survey design to evaluate workshop impact and delivery.

### 2.2 Development of the MPharm-adapted intervention

The intervention evaluated in this study was an adapted version of the pre-existing *Prepare, Support, Prevent* (PSP) module.^24^ PSP is an evidence-informed suicide prevention module for undergraduate health and social care students. Commissioned by the Health Service Executive National Office for Suicide Revention (HSE NOSP) and developed by the National Suicide Research Foundation (NSRF) using a stakeholder-engaged process, it is typically delivered in four two-hour weekly sessions. Topics covered include (i) the epidemiology of suicide, (ii) risk and protective factors (iii) effective communication and (iv) safety planning. Students' personal wellbeing is also incorporated.

For the purposes of this cohort, PSP was adapted to align specifically with the learning needs of pharmacy students. While the original PSP course was developed by a national multi-disciplinary stakeholder group of healthcare academics, course content was adapted for this workshop by an established pharmacy academic working in suicide prevention education. The workshop comprised of a single three-hour session, scheduled for two groups of approximately 30 students per group. Prior to attending the in-person workshop, students were asked to complete “*Let's Talk About Suicide*” – a ninety-minute online training programme developed by the Health Service Executive Ireland available to the public.^25^ This served to provide a basic overview of suicide prevention prior to exploring the topic from the pharmacist's perspective.

Workshop content, case examples, and group discussion, were focused on the pharmacist's scope of practice, while retaining the core principles of suicide prevention education for health and social care students more generally. Case material was contextualised to pharmacy practice, illustrating typical pharmacy-based encounters, initiation of conversations about suicide, medicines-related concerns, and interactions with concerned family members. Emphasis was placed on pharmacists' accessibility as frontline healthcare professionals, and their unique therapeutic relationships with those who may be at risk of suicide.

The session was co-facilitated by two experienced facilitators. Facilitator 1 was the aforementioned pharmacy academic and experienced suicide prevention researcher who holds a postgraduate diploma in Teaching and Learning in Higher Education. Facilitator 2 was also a suicide prevention researcher with previous pedagogical training. Students received recognition of attendance through a certificate of completion.

### 2.3 Study setting and eligibility

The study was conducted at University College Corkwithin the fifth and final-year pharmacy programme (MPharm). The MPharm is a level 9 on the Irish National Framework of Qualifications. It follows a previous four-year bachelor's in pharmacy degree, and all five years are required for pharmacy registration in Ireland. A three-hour suicide prevention workshop was embedded within the Applied Clinical Pharmacy module as part of the existing curriculum, and all students enrolled in the programme were scheduled to attend the workshop as part of their course timetable.

Participation in the research component was voluntary, and students could attend the curricular workshop without taking part in the research study without academic disadvantage. Eligibility for participation included that students be aged 18 years or over, enrolled in the MPharm programme and able to provide informed consent.

### 2.4 Recruitment strategy

Students were provided with a Participant Information Leaflet inviting them to contribute to the evaluation of the workshop. Students who wished to participate in the research component registered through a secure Qualtrics link, where they were required to provide informed consent prior to completing a baseline survey. Students were informed that they could withdraw from the research at any time without penalty and without affecting their ability to attend or complete the educational workshop. This approach was adopted to ensure a clear distinction between compulsory curricular attendance and optional participation in the research evaluation.

### 2.5 Safety considerations

Given the sensitive nature of suicide-related content, a safety protocol developed by the NSRF and informed by extensive suicide prevention training expertise was implemented to support student wellbeing throughout delivery of the workshop. Prior to the session, students were informed in advance of the potentially distressing subject matter. Students were also advised that, should they prefer not to participate, they could contact the Module Lead to discuss this confidentially, with no academic penalty or adverse consequences.

At the beginning of the workshop, facilitators again acknowledged the sensitive nature of the content, encouraged students to engage at their own pace, and advised that students could step out temporarily at any stage if required. Information on available student wellbeing and support services was provided in advance and reiterated during and after the session.

### 2.6 Ethical approval

Ethical approval for the study was obtained from the [UCC Social Research Ethics Committee] (Log 2024–158).

### 2.7 Workshop evaluation

A pre-post workshop survey was administered via Qualtrics to evaluate the educational intervention. The baseline survey (T1) was made available online for completion prior to engagement with the suicide prevention content, and the post-module survey (T2) was administered at the end of workshop. Quantitative data were collected using two established structured questionnaires which were aligned with the workshop's intended learning outcomes.

#### 2.7.1 Suicide attitudes, behaviours and competence (SABC) scale

Students' self-reported suicide prevention attitudes, behaviours, and competence were assessed using the relevant subscales from the Suicide Attitudes, Behaviours and Competence (SABC) Scale.^26^ For the purpose of this evaluation, T1 included the importance, behaviour and competence subscales, while T2 only measured importance and competence due to lack of sufficient time to demonstrate behavioural change. The SABC measures seven key aspects of suicide prevention (e.g. talking to someone who may be at risk of suicide about their thoughts and feelings; listening empathetically to a person with suicidal thoughts). These aspects are assessed across three subscales (i) importance – the participant's perceived importance of each aspect; (ii) behaviour – how frequently the participant has engaged in each of these aspects in the preceding four weeks; and (iii) competence – the participant's perceived competence in performing each of these aspects. Items from each subscale were rated 1–5 on Likert scales, with higher scores indicating greater perceived importance, more frequent behaviour, or higher perceived competence respectively. Possible total scores for each subscale ranged from 7 to 35.

Although the SABC has not undergone formal psychometric validation, it has been used previously in comparable populations to evaluate suicide prevention education and training outcomes.^26^, ^27^

#### 2.7.2 Acceptability, feasibility and appropriateness scale (AFAS)

The Acceptability, Feasibility, and Appropriateness Scale (AFAS) was used to evaluate participants' perceptions of the workshop.^28^

The AFAS is a structured self-report measure designed to assess key implementation outcomes relevant to the uptake and integration of new educational or behavioural interventions.^28^ It comprises of 14 items across three domains. The appropriateness subscale (6 items) assesses the perceived relevance, suitability, and fit of the intervention; the feasibility subscale (3 items), assesses the perceived practicality and ease of participation in the intervention; and the acceptability subscale (5 items), assess participants' overall satisfaction with and approval of the intervention. Each item is rated on a five-point Likert scale ranging from 1 (not at all) to 5 (extremely), with higher scores indicating more favourable perceptions of the intervention. The AFAS has demonstrated good psychometric properties in comparable educational and professional training interventions.^27^, ^29^ Whilst to our knowledge this is the first use of this scale in pharmacy education research, it has been previously employed to evaluate interprofessional undergraduate suicide prevention education which included pharmacy student participants.^27^ The scale has also been used in the evaluation of training for community-based mental health service providers.^28^ An abbreviated version was used by a recent study to measure the quality of the Collaborative Assessment and Management of Suicidality (CAMS) training in mental health and healthcare professionals.^29^

#### 2.7.3 Additional measures

Four additional individual questions were posed to capture information regarding (i) continued workshop inclusion in the MPharm programme; (ii) engagement with the online pre-learning; (iii) preferred timing of workshop offering within the programme and (iv) the inclusion of student personal wellbeing content.

A final free-text response box was also included within the T2 survey to offer participants the opportunity to provide additional feedback on the workshop. However, engagement with this question was limited, therefore these data were not included in the present analysis.

### 2.8 Data analysis

Quantitative data were analysed using IBM SPSS Statistics (version 29). Descriptive statistics were used to summarise participant demographics and baseline characteristics. Continuous variables were reported using mean (M) and standard deviation (SD), while categorical variables were reported as frequencies and percentages.

Changes in matched continuous variable pre-post responses were examined using paired-sample *t*-tests i.e., the SABC importance and competence subscale scores. Mean differences, 95% confidence intervals (CIs), and *p*-values were reported. Effect sizes were calculated using Cohen's *d*, with values of 0.2, 0.5, and 0.8 conventionally interpreted as small, medium, and large effects, respectively.^30^ Statistical significance was set at *p* < 0.05. The AFAS scale was analysed descriptively using frequencies, percentages, and summary subscale scores. Internal reliability of both the SABC and AFAS scales was examined using Cronbach's alpha coefficients.^31^

Where one item was missing in an SABC subscale, scores were calculated by dividing the completed item total by the number of questions answered and then multiplying by the number of questions in the sub-scale (i.e. divided by 6 and multiplied by 7). Cases with more than one missing item within a subscale were to be excluded from the analyses. For AFAS subscales, equivalent scoring procedures were applied. Cases with more than one missing item within any AFAS subscale were to be excluded from the analyses.

## 3 Results

Of the 60 students who were enrolled in the module, 23 and 30 attended the first and second workshop respectively (*n* = 53). A total of 26 students completed the baseline survey (26/53, 49% response rate) and were included in the descriptive demographic analysis. An additional student completed the AFAS scale (*n* = 27) in the post-workshop survey and was included in the analysis.

### 3.1 Demographics

Most participants identified as female (*n* = 19, 73.1%) and were aged 18–24 years (*n* = 25, 96.2%). Only one participant (3.8%) reported having completed previous suicide prevention training, which was undertaken independently as an online course in a personal capacity. Five participants (19.2%) reported having encountered somebody at risk of suicide within a work or placement setting. Supplementary free-text responses to this question indicated that these encounters mostly occurred in community pharmacy settings and involved disclosures of self-harm thoughts, previous suicide attempts, acute mental health crises, or concerns raised during contact with vulnerable patients. One respondent described a patient receiving methadone treatment who presented to the pharmacy after reporting suicidal thoughts in an emergency department setting.

### 3.2 Suicide prevention attitudes, behaviours and competence

A total of 26 participants completed the SABC Scale. Students reported infrequent performance of key suicide prevention behaviours (M = 8.27, SD = 2.55) in the four weeks prior to completing the T1 survey.

**Fig. 1:**
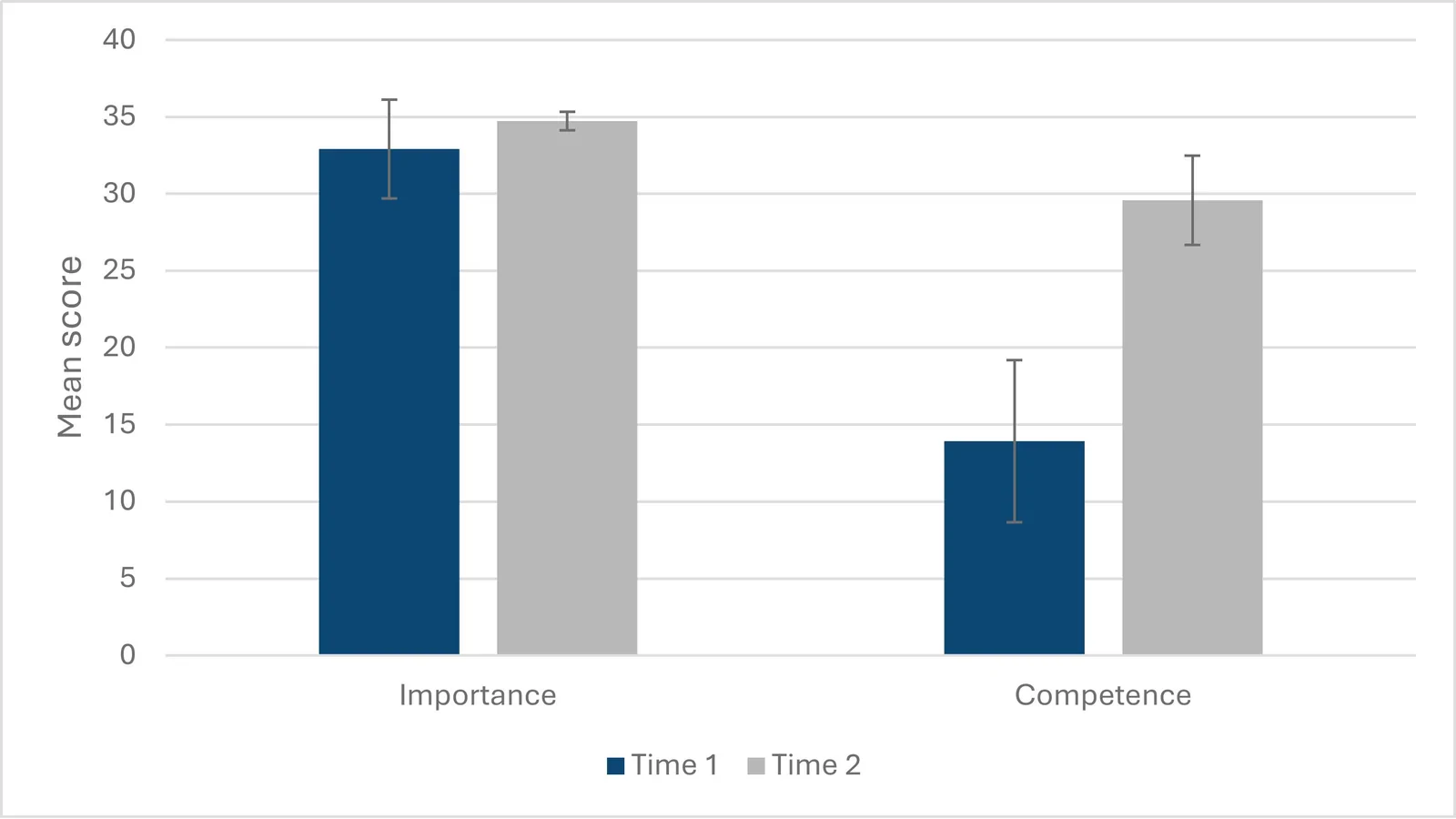
Changes in mean perceived suicide prevention importance and competence scores (T1 vs T2).

Paired-sample *t*-tests of importance and competence subscales indicated improvements in mean scores following participation in the workshop. Scores on the SABC importance subscale increased from T1 (M = 32.91, SD = 3.22) to T2 (M = 34.73, SD = 0.60), representing a mean increase of 1.82 (95% CI: 0.66, 2.98, *p* = 0.004), with a moderate effect size (Cohen's *d* = 0.63). The SABC importance subscale demonstrated good internal consistency (Cronbach's α = 0.82). Scores on the SABC competence subscale also increased from T1 (M = 13.92, SD = 5.28) to T2 (M = 29.58, SD = 2.90), representing a mean increase of 15.65 (95% CI 13.30,18.00, *p* < 0.001), with a large effect size (Cohen's *d* = 2.69). These findings are illustrated in Fig. 1. The SABC competence subscale also demonstrated good internal consistency (Cronbach's α = 0.86).

### 3.3 Workshop acceptability, appropriateness and feasibility

Post-intervention survey responses indicated favourable perceptions of the workshop across all AFAS domains (*n* = 27). Mean acceptability scores were high (M = 28.81/30, SD = 1.80), representing 96.0% of the maximum possible score. Mean feasibility scores were similarly favourable (M = 13.56/15, SD = 1.67), representing 90.4% of the maximum possible score. The appropriateness of the workshop also scored highly (M = 21.78/25, SD = 2.99), representing 87.1% of the maximum possible score. All AFAS subscales demonstrated acceptable internal consistency (Cronbach's α = 0.70–0.86). A full item breakdown of the AFAS findings are presented in Table 1.

**Table 1:** Course acceptability, feasibility and appropriateness scale (AFAS) scores.

Item	Mean score	SD	Range (min to max)	Cronbach alpha
**Acceptability (maximum score 30)** **(*n*** **=** **27)**	**28.81**	**1.80**	**22–30**	**0.79**
1. To what extent are you satisfied with the training you received, and the practices covered?	4.78	0.51	3–5	
2. How credible did you find the facilitators?	4.96	0.19	4–5	
3. How well organised and executed do you believe the training program to be?	4.78	0.42	4–5	
4. How satisfied are you with the content of the training and the practices covered?	4.89	0.42	3–5	
5. How satisfied are you with the complexity of the training and the practices covered?	4.85	0.36	4–5	
6. How comfortable are you with the practices contained within the training?	4.56	0.58	3–5	
**Feasibility (maximum score 15)** **(n** **=** **27)**	**13.56**	**1.67**	**9–15**	**0.70**
7. How useful are the information and practices from the training to you in your future clinical practice?	4.78	0.51	3–5	
8. To what extent do you expect to be able to incorporate the concepts and techniques from the training into your daily work activities?	4.26	0.86	2–5	
9. How compatible do you expect the practices from the training to be with the practical realities and resources of your health/social care setting?	4.52	0.70	3–5	
**Appropriateness (maximum score 25)** **(n** **=** **27)**	**21.78**	**2.99**	**17–25**	**0.86**
10. How compatible are the information and practices with your health/social care setting's mission or service provision mandate?	4.52	0.58	3–5	
11. How relevant are the information and practices to your patient population?	4.52	0.70	3–5	
12. How well do the information and practices fit with your current treatment modality, theoretical orientation, or skill set?	4.44	0.64	3–5	
13. How compatible are the information and practices with your workflow timing (e.g., when and for how long you see patients)?	3.85	1.03	2–5	
14. How well do the information and practices from the training fit with your overall approach to service delivery and the setting in which you provide care?	4.44	0.70	3–5	

### 3.4 Future curriculum integration and student feedback

All respondents (*n* = 26) recommended that suicide prevention content should continue to be embedded within the MPharm programme. Of these, 57.7% (*n* = 15) supported continuation in its current form, while 42.3% (*n* = 11) indicated that the content should continue, with scope for further expansion. Among students who responded to the item regarding timing of delivery (*n* = 25), 72.0% (*n* = 18) reported that they would have liked to receive suicide prevention training earlier in their degree programme, 16.0% (*n* = 4) indicated that they would not like to receive this training earlier, and 12.0% (*n* = 3) were neutral.

Regarding the inclusion of student wellbeing content within the workshop (*n* = 25), 84.0% (*n* = 21) found this helpful, while a further 12.0% (*n* = 3) reported that they found it helpful but would have welcomed expansion. One participant (4.0%) indicated that they did not find this component helpful, with no further explanation given.

## 4 Discussion

This study provides encouraging preliminary evidence that suicide prevention education can be successfully embedded within an MPharm curriculum in a way that is acceptable, feasible, appropriate and educationally valuable to students. Participation in the workshop saw improvements in self-reported competence across core suicide prevention actions, alongside smaller gains in the perceived importance of these actions. Students also rated the intervention highly across implementation outcomes, with universal support among respondents for continued inclusion of this content within the programme. These findings suggest that suicide prevention training is valued by pharmacy students and can be integrated into core pharmacy curricula in a manner that is both educationally effective and practically deliverable.

A key finding of the present study was the improvement in perceived competence following participation in the workshop. This is particularly relevant in the context of pharmacy education, where students may encounter individuals experiencing suicidal distress during placements and, later, in professional practice, yet may receive limited formal preparation for such situations.^8^ The Pharmaceutical Society of Ireland (PSI) Core Competency Framework for Pharmacists^32^ underpins pharmacy education, experiential learning and continuing professional development. Although suicide prevention is not explicitly mentioned, it does strongly emphasise expectations relating to person-centred care, communication, professional judgement and patient safety. Pharmacists, particularly those working in community settings, are among the most accessible and trusted healthcare professionals^10^ and may be approached by individuals experiencing mental distress, crisis, or suicidal thoughts. In such scenarios the aforementioned competencies are key in providing appropriate support.

Pharmacists therefore have an important role in recognising concern, responding supportively, and facilitating onward help-seeking. However, previous Irish research suggests that many pharmacy staff feel underprepared for this aspect of practice. O'Driscoll et al. (2023) reported that community pharmacists and pharmacy staff frequently encountered patients presenting with suicide risk, self-harm, or significant psychological distress, yet many respondents reported feeling underprepared to manage such interactions.^8^ Most participants had not previously received suicide prevention training, despite regular exposure to these presentations. Respondents described uncertainty regarding how best to communicate with distressed patients, concerns about referral pathways, and a lack of confidence in responding appropriately.^8^ These findings highlight a clear gap between the realities of pharmacy practice and the training historically available to the profession. In this regard, the competence gains observed in the present study are notable and suggest that structured, role-relevant suicide prevention education may help to bridge the gap between broad professional competency expectations and the specific interpersonal, safety-related and referral skills required when responding to suicidal distress in pharmacy settings.

The findings are also consistent with a growing international evidence base relating to suicide prevention in pharmacy education. Studies involving pharmacy students have reported improvements in suicide-related knowledge, confidence, and willingness to intervene following brief optional educational interventions, including gatekeeper training and Mental Health First Aid-informed approaches.^18^, ^33^, ^34^, ^35^ Similar benefits have been observed among qualified pharmacists, where training has been associated with increased confidence, improved attitudes and greater readiness to engage with at-risk individuals.^20^, ^34^, ^36^ In addition, pharmacy students appear to recognise the relevance of suicide prevention to their future professional role. For example, a study by Mospan and Gillette (2020), reported that 79% (*n* = 57) of first-year pharmacy students agreed or strongly agreed that they have a professional responsibility to assess suicidal ideation.^37^ This aligns with the present study, where students rated the workshop as highly acceptable, feasible and appropriate, suggesting that suicide prevention education was perceived as relevant and suitable when embedded into pharmacy training. This literature suggests that pharmacy learners and practitioners can benefit from targeted suicide prevention education, particularly where training includes practical communication strategies and clear referral guidance.

### 4.1 Limitations

This study should be interpreted in light of its limitations. The sample was drawn from a single institution with a small sample size and modest response rate reported, factors which need to be considered in the interpretation and generalisability of the findings.

Outcomes relied on self-report measures of short-term competence, which may not fully reflect behavioural change in practice. Future work could employ the use of vignettes or other formative assessment to help determine student competence gains. Furthermore, the absence of a control group meant that improvements could not be attributed solely to the intervention with certainty. Follow-up data were collected shortly after delivery, so longer-term retention of gains and application of learning in practice remains unknown. Finally, participants who completed evaluation measures may have been more engaged or positively disposed toward the topic than the general student population.

Despite these limitations, this study contributes important evidence regarding suicide prevention training within pre-registration pharmacy education. In particular, the use of the AFAS and SABC scales in this cohort is a novel approach to determining suicide prevention workshop effectiveness and pharmacy student satisfaction with such training.

Given pharmacists' accessibility, trusted status, and potential contact with individuals experiencing suicidal crisis, strengthening undergraduate preparation in this area represents a logical and potentially impactful workforce development strategy.

Future research should examine longer-term retention of competence and the translation to placement and professional practice behaviours. Multi-site studies across pharmacy schools would also help to establish broader feasibility and inform development of national educational and competency standards for suicide prevention education within pharmacy training.

## 5 Conclusions

This study demonstrates that suicide prevention education improves pharmacy students' perceived readiness to respond to individuals at risk of suicide. It adds to the large body of evidence supporting suicide prevention training in pharmacy education and provides additional evidence that such training can serve as an effective initial exposure for pharmacy students preparing for practice. Students viewed the workshop positively and strongly supported its continued inclusion within the curriculum. Furthermore, the use of the SABC scale and AFAS is a valuable development in the evaluation of pharmacy education interventions, including consideration of key implementation outcomes such as acceptability, feasibility, and appropriateness. Given the accessibility of pharmacists and their potential role in supporting individuals in distress, suicide prevention education represents an important consideration for contemporary pharmacy curricula.

## CRediT authorship contribution statement

**Michelle O'Driscoll:** Writing – review & editing, Writing – original draft, Visualization, Validation, Supervision, Project administration, Methodology, Investigation, Funding acquisition, Formal analysis, Data curation, Conceptualization. **Kerrie Gallagher:** Writing – review & editing, Writing – original draft, Project administration, Methodology, Formal analysis, Data curation, Conceptualization. **Maria Donovan:** Writing – review & editing, Project administration, Methodology, Conceptualization. **Laura Sahm:** Writing – review & editing, Validation, Supervision, Project administration, Methodology. **Aoife Fleming:** Writing – review & editing, Validation, Supervision, Project administration, Methodology. **Clíodhna O'Brien:** Writing – review & editing, Validation, Methodology, Formal analysis. **Ailish O'Neill:** Writing – review & editing, Supervision, Resources, Project administration, Conceptualization. **Eve Griffin:** Writing – review & editing, Visualization, Validation, Supervision, Methodology, Funding acquisition, Conceptualization.

## Funding statement

This project was funded by the Health Service Executive National Office for Suicide Prevention (HSE NOSP).

## Declaration of competing interest

Ailish O'Neill is employed by the funder (HSE NOSP). She was not involved in data collection and analysis and only advised on manuscript drafting, editing and reviewing.

## References

[1] World Health Organisation (2021). https://iris.who.int/bitstream/handle/10665/341728/9789240026643-eng.pdf.

[2] Xie L., Tang L., Liu Y., Dong Z., Zhang X. (2025). Global burden and trends of self-harm from 1990 to 2021, with predictions to 2050. Front Public Health.

[3] Purebl G., Petrea I., Shields L. (2015). https://health.ec.europa.eu/document/download/ab5ab6a0-b8a5-47a7-9c81-91a0183c3a19_en.

[4] Ahmedani B.K., Westphal J., Autio K. (2019). Variation in patterns of health care before suicide: a population case-control study. Prev Med.

[5] Leahy D., Larkin C., Leahy D. (2020). The mental and physical health profile of people who died by suicide: findings from the suicide support and information system. Soc Psychiatry Psychiatr Epidemiol.

[6] El-den S., Collins J.C., Chen T.F., O’Reilly C.L. (2021). Pharmacists’ roles in mental healthcare: past, present and future. Pharm Pract (Granada).

[7] Royal Pharmaceutical Society (2018).

[8] O’Driscoll M., Carpenter D.M., Foley A., Moloney E., Reddin K., Sahm L.J. (2023). A needs assessment for suicide prevention training within community pharmacies. Explor Res Clin Soc Pharm.

[9] Witry M.J., Carpenter D.M. (2023). Community pharmacist encounters with patients displaying suicide warning signs: a cross-sectional survey. J Am Pharm Assoc.

[10] Ipsos (2025). https://www.ipsosbanda.ie/ipsos-ba-veracity-index-2025-who-do-we-trust-in-ireland/.

[11] Kassir H., Eaton H., Ferguson M., Procter N.G. (2019). Role of the pharmacist in suicide prevention: primely positioned to intervene. Pharm Pract Res.

[12] Kamal L., Jacob S.A. (2023). Pharmacists’ experiences, perceptions, and attitudes towards suicide and suicide prevention: a scoping review. Pharmacy (Basel).

[13] O’ Brien C., Gallagher K., O’ Driscoll M. (2025). Suicide prevention curriculum development for health and social care students: a scoping review. PloS One.

[14] World Health Organization (2021). Live Life: An Implementation Guide for Suicide Prevention in Countries.

[15] O’Driscoll M., Gallagher K., O’Brien C., Corcoran P., Griffin E. (2026). Implementing a suicide prevention module in undergraduate health and social care education: insights from a World Café and online survey. J Ment Health Train Educ Pract.

[16] Pothireddy N., Lavigne J.E., Groman A.S., Carpenter D.M. (2022). Developing and evaluating a module to teach suicide prevention communication skills to student pharmacists. Curr Pharm Teach Learn.

[17] Stover A., Lavigne J., Carpenter D. (2023). A scoping review of suicide prevention training programs for pharmacists and student pharmacists. Am J Pharm Educ.

[18] Gorton H.C., Elliott R., Noonan I. (2021). Student pharmacists and mental health nurses training together in suicide prevention: an evaluation of interprofessional education. Int J Pharm Pract.

[19] Willson M.N., Robinson J.D., McKeirnan K.C., Akers J.M., Buchman C.R. (2020). Training student pharmacists in suicide awareness and prevention. Am J Pharm Educ.

[20] Painter N.A., Kuo G.M., Collins S.P., Palomino Y.L., Lee K.C. (2018). Pharmacist training in suicide prevention. J Am Pharm Assoc.

[21] Stover A.N., Lavigne J.E., Shook A., MacAllister C., Cross W.F., Carpenter D.M. (2023). Development of the pharm-SAVES educational module for gatekeeper suicide prevention training for community pharmacy staff. Health Expect.

[22] Department of Health (2020). Sharing the Vision: A Mental Health Policy for Everyone. https://assets.gov.ie/static/documents/sharing-the-vision-a-mental-health-policy-for-everyone.pdf.

[23] National Office for Suicide Prevention (2015).

[24] O’Driscoll M., Gallagher K., O’Brien C. (2026).

[25] Health Service Executive, National Office fpr Suicide Prevention (2026). Let’s Talk About Suicide. Let’s Talk About Suicide. https://traininghub.nosp.ie/.

[26] Arensman E., Coffey C., Griffin E. (2016). Effectiveness of depression–suicidal behaviour gatekeeper training among police officers in three European regions: outcomes of the optimising suicide prevention programmes and their implementation in Europe (OSPI-Europe) study. Int J Soc Psychiatry.

[27] O’Driscoll M., Gallagher K., Magner C. (2026).

[28] Cho E., Lyon A.R., Tugendrajch S.K., Marriott B.R., Hawley K.M. (2022). Assessing provider perceptions of training: initial evaluation of the acceptability, feasibility, and appropriateness scale. Implement Res Pract.

[29] Norrod P.E., MacDonald M., Link K., Ickes M.J. (2025). Evaluating implementation of the collaborative assessment and management of suicidality (CAMS) training for rural suicide prevention among mental health and healthcare professionals. Issues Ment Health Nurs.

[30] Cohen J. (2013).

[31] Cronbach L.J. (1951). Coefficient alpha and the internal structure of tests. Psychometrika.

[32] The Pharmaceutical Society of Ireland (2022). Core Competency Framework for Pharmacists. https://www.psi.ie/sites/default/files/2024-06/PSI_Core_Competency_Framework.pdf.

[33] O’Reilly C.L., Bell J.S., Kelly P.J., Chen T.F. (2011). Impact of mental health first aid training on pharmacy students’ knowledge, attitudes and self-reported behaviour: a controlled trial. Aust N Z J Psychiatry.

[34] Frond D., Habba S., Stewart B., Burghardt K.J. (2025). The effect of mental health first aid training on pharmacist and pharmacy student confidence and knowledge: a systematic review and meta-analysis. Brain Sci.

[35] Robinson J.D., Maslo T.E., McKeirnan K.C., Kim A.P., Brand-Eubanks D.C. (2020). The impact of a mental health course elective on student pharmacist attitudes. Curr Pharm Teach Learn.

[36] Carpenter D.M., Stover A., Shackley A.G. (2026). The impact of an online suicide prevention training program on community pharmacy staff outcomes: a cluster randomized trial. J Am Pharm Assoc.

[37] Mospan C.M., Gillette C. (2020). Student pharmacists’ attitudes toward suicide and the perceived role of community pharmacists in suicidal ideation assessment. Am J Pharm Educ.

